# What makes women with food hypersensitivity do self-management work?

**DOI:** 10.1186/s12913-019-4243-6

**Published:** 2019-07-08

**Authors:** Monika Dybdahl Jakobsen, Aud Obstfelder, Tonje Braaten, Birgit Abelsen

**Affiliations:** 1 Department of Community Medicine, UiT The Arctic University of Norway, Tromsø, Norway; 2 Center for Care Research, The Norwegian University of Science and Technology (NTNU), Gjøvik, Norway; 3 Department of Health and Care Sciences, UiT The Arctic University of Norway, Tromsø, Norway; 4 Norwegian Centre for Rural Medicine, Department of Community Medicine, UiT The Arctic University of Norway, Tromsø, Norway

**Keywords:** Food hypersensitivity, Chronic conditions, Motivation, Resources, Conservation of resources (COR) theory, Self-determination theory

## Abstract

**Background:**

Managing a chronic condition takes work, and it is considered important that patients carry out this work. However, knowledge is lacking on what elements enhance self-management work.

Persons with food hypersensitivity (FH) seem to do self-management work despite the relatively little support they receive. Our aim is to explore what makes women with FH carry out the work of managing their condition. Our research will shed light on the health care needs of women with FH and contribute to the knowledge on self-management among persons with chronic conditions.

**Methods:**

We used the Self-determination theory and the Conservation of resources theory to analyze 16 qualitative individual interviews with women with FH aged 39–67 years.

**Results:**

Our participants reported that eating selected foods resulted in uncomfortable symptoms, and their main motivation for carrying out self-management work was the wish to avoid these symptoms and their consequences.

Participants’ individual resources were crucial to the management of FH, and those who had a social network that included people with relevant competencies clearly benefited from this.

Hindrances to the management of FH included competing priorities and not wanting to break with the social expectation of sharing a meal.

**Conclusions:**

Women with FH carried out self-management work because they were highly motivated. Important motivators included the uncomfortable symptoms that resulted from consuming some foods, which had negative consequences on their lives or could bring shame. The ability to perform self-management work was dependent on the availability of individual and social resources. Indeed, women with FH who have the individual and social resources necessary to manage their condition may not need health services, whereas those who do not have these resources, or have significant competing priorities, may need assistance from health services.

The desire to avoid uncomfortable symptoms can be a motivator for persons with chronic conditions to do self-management work, while a lack of symptoms can reduce motivation. The competing role of basic needs can take two forms: when fulfilled, these needs may contribute to self-management work; however, people may opt out of self-management in order to fulfil basic needs.

## Background

The number of persons with chronic conditions is increasing [1, 2]. This presents a challenging and costly problem for health services, which tend to assign more and more of the work of managing such conditions to the patient [3]. It is considered important that patients do this work, both because it is important for their health and because it lessens the burden on the health care system [4–6]. However, patients do not always conduct this work, which is seen as a major problem and a growing concern [5]. Studies have tried to illuminate what may increase self-management work among patients, but knowledge is still lacking [5].

However, studies do argue that the degree to which self-management work is done depends on the patient’s degree of motivation [6]. According to the Self-determination theory (SDT), high-quality motivation requires patents to internalize values and skills for change [7], and high-quality motivation is more likely to be achieved if three basic psychological needs are satisfied: the need to be 1) competent, 2) autonomous, and 3) related to others [7, 8]. The quality of motivation is also influenced by the goals that are set. For example, intrinsic goals like personal growth and health may result in higher-quality motivation than extrinsic goals such as wealth [7].

While SDT addresses the elements that contribute to high-quality motivation, the Conservation of resources (COR) theory addresses the actual motivators, and discusses how resources and external conditions influence our ability to do what we are motivated to do. The basic tenet in COR theory is that people use available resources to retain, foster, and protect the things they value [9, 10], such as peace, family, self-preservation, well-being, a positive sense of self, and health [9:228]. To protect these things, we use different resources, including material, personal (e.g. skills), and social resources [9]. These resources are strongly associated. For example, individual resources like self-esteem, self-efficacy, and optimism are correlated with social support. Thus, individual resources and social support run in ‘packs’, referred to as ‘resource caravans’ [9]. According to COR theory, the loss or gain of resources can trigger negative or positive resource cycles: people who lack or lose resources are more vulnerable to further resource loss, and achievement of new resources can start positive processes that lead to further resource achievement [11].

According to COR theory, conditions that are external to the individual can also influence people’s ability to protect the things they value; these external conditions are called ‘resource caravan passageways’ [9]. Supportive resource caravan passageways enhance people’s resource reservoirs, and thus their ability to protect what they value. Physical safety, clean water, and good schools are examples of caravan passageways that support people’s resource reservoirs [9]. Supportive health care providers (both systems and practitioners) who mitigate treatment burdens can also be seen as supportive resource caravan passageways [12]. However, resource caravan passageways can also obstruct people’s resource reservoirs, and in this way people who struggle to protect their resources can be hindered by conditions beyond their control [9]. Hobfoll [11] also emphasized that persons belonging to higher social layers are more likely to have caravan passageways that support or enhance their resources. Thus, although it is considered highly important that patients do self-management work for their chronic conditions [6], motivation, available resources (resource caravans) and external conditions (resource caravan passageways) may influence the extent to which this work is actually done.

One group that seems to do self-management work is persons with food hypersensitivity (FH). (FH is a collective term for all non-toxic adverse reactions to foods, and is also referred to as food allergies and food intolerances [13]). In this study, individuals with FH include all those who report that they are hypersensitive to foods; some of them have received a diagnosis of FH from conventional medicine, others have self-defined or alternative medicine-defined FH. Persons with FH carry out self-management work despite the fact that this work can be socially problematic, and despite sparse health services [14–16]. Indeed, FH is controversial; there have been suggestions that too many people claim to have FH, and there is a possible stigma attached to having a restricted diet [17–19]. Self-management work related to FH may include finding out which foods cause symptoms, purchasing and making foods that do not include symptom-causing components, instructing other persons on the preparation of foods or clarifying the content of meals with other persons, maintaining a nutritious diet despite restrictions, and avoiding a diet that is too restricted.

Our aim is to explore what makes adult women with FH carry out the work of managing their condition. This study is the product of a project that includes only adult women and thus is restricted to this group. Our research will shed light on the health care needs of women with FH and may contribute to the knowledge on self-management among persons with chronic conditions.

## Methods

### Design and sample

We analyzed 16 qualitative individual interviews of women with FH aged 39–67 years (mean age 49.7 years). MJ conducted the practical work of recruitment, interviewing, and analyzing, in close cooperation with the coauthors. The Norwegian Center for Research Data was notified about the study, as per current standards.

The interviews were semi-structured and were planned and carried out for the initial purpose of illuminating the nature of the work that goes into managing FH (as described in another article in press [20]). MJ developed the interview guide, and the guide was tested in a pilot interview with a woman with FH. This pilot interview led to some small amendments in the interview guide, as well as some amendments in the interview technique.

However, after the first couple of interviews with study participants, the research team noticed that the interviewees showed a considerable ability to manage their FH. This roused the authors’ curiosity, and it was decided that that the interviewer should ensure that the analytical question “What is it that makes them manage FH?” was illuminated in the interviews.

The study sample was chosen purposively, with the intention to interview women with and without a diagnosis of FH and with and without other chronic health conditions. Most participants were recruited through acquaintances of MJ or through contacts at The Norwegian Asthma and Allergy Association and The Norwegian Celiac Association. These contacts outside the research team gave potential interviewees information about the study, asked whether they wanted to participate in the study, and asked whether MJ could contact them. This recruitment procedure was chosen to prevent potential interviewees from feeling pressured to participate, under the assumption that a request from someone outside the research team would place less pressure on potential interviewees than a direct request from the researchers. Furthermore, since we placed strong emphasis on the fact that participation was voluntary, women did not have to explain any unwillingness to participate. Thus, we do not know why some women chose not to participate.

In addition to the described recruitment strategy, one woman was recruited through an invitation posted on the Norwegian Asthma and Allergy Associations Facebook page, and one woman self-recruited when she heard about the project topic from MJ.

All scheduled interviews were carried out as planned, except one that was canceled for reasons unrelated to the project. A new participant was recruited to replace this participant.

### Data collection and analysis

The interviews were carried out in five different towns in Norway between August and November of 2016. All interviews were conducted face-to-face in an undisturbed, quiet location chosen by the participant; in the interviewees’ homes, in the interviewees’ place of work, or on the premises of UiT The arctic university of Norway. One interview per participant was conducted, and only the interviewer and the individual interviewee were present.

Before the interviews started, the participant signed a written informed consent form, and all interviews began with the interviewer asking the participant to describe how she found out that there are food(s) she cannot tolerate. Most participants gave thorough answers to this question, and the interviewer used this information to pose follow-up questions concerning the work of managing FH and what made the participants manage FH. At the end of the interview, the interviewer looked through the interview guide to make sure all topics had been covered. The interviews lasted from 53 to 98 min (mean 67 min), and were audio recorded and transcribed by a professional transcriber. The participants were encouraged to contact the interviewer after the interview if they had anything they wanted to add to the interview, and one participant did so. The participants were not asked to give feedback on the findings.

Before the analysis, the interviews were listened to and read through to allow for familiarization with the data. Then all interviews were read through again, with the focus on exploring what made the women manage FH. During this process, two important answers to this question were discovered: (1) The interviewees showed significant *motivation* to do the work of managing FH, and (2) The interviewees had *ability* to do the work of managing FH. Consequently, further analyses focused on these two aspects.

First MJ read the interviews, noted what motivated each participant, and made a condensation, which is presented in the second part of the results section. COR theory and SDT were used to interpret these findings and understand the participants’ motivations.

Secondly, the researchers analyzed what made the participant able to do the work of managing FH. After having read the interviews we had the clear impression that the participants’ individual and social resources, as well as external factors, were important to their ability to do self-management work. This aspect is also emphasized in COR theory, and thus we found COR theory concepts of ‘resource caravans’, ‘caravan passageways’, and ‘resource spirals’ to be useful when discussing the findings. SDT was also used to discuss and understand the findings, and SDT’s focus on the interrelationship between motivation and the basic psychological needs of autonomy, competence, and relatedness in particular influenced our interpretation of the interviews. We did not use any software in the analysis.

We observed that the main motivators, as well as the factors that made participants able to do the work of managing FH, were mentioned within the first seven interviews, while in the remainder of the interviews these themes were elaborated and illuminated from other angles. This indicates that saturation was achieved within the 16 interviews, which is in line with studies that have concluded that saturation in studies with purposive samples often occur within the first 12 interviews [21].

## Results

### Characteristics of the participants

Sixteen women with FH aged 39–67 years (mean age 49.7 years) participated in this study. Eight of these women had a diagnosis of FH based on conventional medicine tests (food allergies, celiac disease, and/or lactose intolerance), two had a diagnosis of irritable bowel syndrome, two said that a medical doctor had mentioned that they had or may have irritable bowel syndrome, and four had no diagnosis of FH from conventional medicine (See Table 1). Most of the women with a diagnosis of FH from conventional medicine reported an additional, undiagnosed FH. Furthermore, eight of the women had other chronic health complaints such as diabetes, hypothyroidism, rheumatoid arthritis, fibromyalgia, or asthma/allergies that required daily medication, while the other eight did not have such conditions. The interviewees described FH or symptoms of FH that had lasted for years or decades. Fourteen women reported hypersensitivity to more than one food; most women reported hypersensitivity to common foods like milk, gluten, or wheat.

**Table 1:** Participant characteristics

	Diagnosis of food hypersensitivity from conventional medicine	Other chronic conditions	Age	Education level
Dina	Yes, Celiac disease	No	39–49	Master
Eline	Yes, Celiac disease	Yes	50–59	Master
Grethe	Yes, Celiac disease	Yes	50–59	Master
Carina	Yes, Celiac disease and lactose intolerance	Yes	60–67	Secondary
Anna	Yes, Lactose intolerance (and irritable bowel syndrome)	Yes	39–49	Secondary
Helen	Yes, food allergies	No	39–49	Master
Mary	Yes, food allergies	No	39–49	Bachelor
Ruth	Yes, food allergies	No	50–59	Bachelor
Frida	Possible irritable bowel syndrome	No	50–59	Master
Lena	Yes, irritable bowel syndrome	No	39–49	Bachelor
Irene	Possible irritable bowel syndrome	Yes	39–49	Bachelor
Kristina	Yes, irritable bowel syndrome	Yes	60–67	Secondary
Sarah	No	No	39–49	Secondary
Jeanette	No	No	50–59	Bachelor
Nina	No	Yes	50–59	Bachelor
Brita	No	Yes	50–59	Master

Nine interviewees had minor children, eight of whom lived with a partner; and seven had adult children, six of whom lived with a partner. Four women had secondary school as their highest completed education, six had a bachelor’s degree, and six women had a master’s degree.

### Motivation

Many interviewees revealed a strong motivation to do self-management work, and this was expressed, among other things, through an extensive effort to find out which foods caused symptoms, and through the continuous attention they paid to avoid those foods. All participants reported that their physical afflictions or symptoms had persisted for a relatively long period. For some, symptoms began in childhood, for others in adulthood. The most mentioned symptoms were stomach pain, diarrhea, constipation, flatulence, nausea, breathing problems, laxity, low energy, hives, and other skin symptoms. All women had taken the initiative to determine what was causing the symptoms, and after getting advice from others, from health services, from alternative medicine actors, or on their own initiative, they removed one or more foods from their diet, i.e. implemented a restricted diet. After doing this they experienced significant symptom reduction. Some reported that they got rid of their affliction completely, while others experienced improved health and reduced symptoms. The women reported that their main motivation for implementing a restricted diet was to eliminate or reduce physical symptoms they described as uncomfortable or intolerable. Brita (no diagnosis) gave an example of uncomfortable symptoms:

*…suddenly I got sick, I got dizzy, I started sweating, I had to get out, and I threw up.*

The women also described that the symptoms had negative consequences on work, general energy level, leisure activities, and quality of life, and the wish to avoid these consequences were strong motivators to do self-management work. The desire to work and take care of children was an especially strong motivator for the interviewees. Jeanette (no diagnosis) verbalized this:

*I had symptoms for a while; for half a year to three-quarters of a year, all the food just went straight through me (…) and I decided this had to stop. I was on the toilet 20 to 30 times a day. When you have these symptoms, it is almost impossible to go to work, although I did. I* want *to go to work (…) So I decided that I have to find a way to function, I have to stay healthy. I have responsibilities, I have children and I have a family.*

The symptoms could also lead to shame or stigma, which participants tried to avoid by reducing or avoiding the symptoms. Nina (no diagnosis) exemplified this with a story on how she fainted due to intense stomach pain after having ingested an adverse food. She experienced this as embarrassing:

*My stomach hurt so, so much (...) and when we were leaving, I fainted and fell on the floor. That is not a fun thing when you are at a restaurant, and it led to a lot of fuss.*

Participants mentioned how stomach problems and diarrhea could lead to flatulence or involuntary defecation, which was experienced as very embarrassing. Furthermore, they emphasized the shame related to stomach troubles, referring to it as an unspeakable topic. One of the interviewees said:

*It is difficult to speak about this topic (…) it leaves you vulnerable, and it is very embarrassing.*

Some women mentioned additional motivations for implementing a restricted diet. For example, a few participants had experienced threatening allergic reactions. Others, having experienced that removing foods made them healthier, tried to remove other foods as well, hoping that this would further reduce symptoms and improve their health. One interviewee perceived certain alternative diets to be particularly healthy and used a restricted diet to avoid weight gain. Some of the women with celiac disease said that a part of their motivation for implementing a gluten-free diet was to avoid sequela.

In summary, the participants said that the main driving force for removing foods from their diet was to avoid symptoms, because these symptoms were uncomfortable and had negative consequences. However, interviewees who did not have strong symptoms showed a lower motivation to consistently implement a restricted diet compared to those with strong symptoms.

### Resources used in self-management work

As mentioned, many interviewees experienced uncomfortable symptoms. They were highly motivated and expended great effort to avoid these symptoms. In this process, their individual resources, often in combination with available social resources, were crucial in finding out what foods caused symptoms and to manage their FH. This was true regardless of whether the women received a diagnosis or whether they had contact with conventional health services or alternative medicine. Interviewees with a diagnosis of celiac disease from conventional medicine received information from health services at the time of diagnosis about how to manage this condition. However, when they experienced new symptoms later on, they used individual resources combined with advice from family or friends to find out what was causing them. Grethe (celiac disease) said:

*It was a period in which I could not understand why I was feeling ill (…) So I tried to find out what was making me feel ill, as I have done several times. (…)*

When faced with these new symptoms, Grethe went through what she had eaten in the preceding days, and combined this with information from a patient organization and from an acquaintance with celiac disease. Through this process, she understood that wheat starch had caused the symptoms.

Interviewees who had a diagnosis of food allergy or lactose intolerance from conventional medicine also had contact with health services at the time of diagnosis, but after that they used individual and social resources to find out what concrete products and meals to avoid, and which to eat to ensure a varied diet. In this process, they described personal interests in foods and tips from friends and family as useful. This was verbalized by Anna (lactose intolerance):

*I am interested in food (…) and I have some friends who…we talk a lot about food and give each other advice.*

Those who did not have any of the above diagnoses from conventional medicine, but received help from actors in alternative medicine, combined this help with individual and social resources. For example, an alternative medicine clinic advised Jeanette (no diagnosis) to start a very restricted diet and then to gradually reintroduce foods. Furthermore, a family member supported her and helped her to interpret tests she underwent to assess whether she was receiving the correct amount of nutrients despite the restricted diet. In addition, Jeanette used her own time and effort to carry out the very restricted diet, and to evaluate how she reacted to newly reintroduced foods.

Some interviewees did not receive help from health services or from alternative medicine. Instead they mainly relied on their own experiences and resources. Some individual resources that were useful were interest in food, food-related education, and a range of problem-focused strategies, such as planning in advance and bringing one’s own food.

One individual resource that seemed crucial to self-management work was the ability to critically assess the advice of others. Interviewees described that some alternative medicine actors recommended diets that were too comprehensive. Other interviewees described that advice from acquaintances was characterized by confusion between FH and other reasons for having a special diet. However, several interviewees critically assessed this information, chose what they considered relevant for them, and thus avoided diets that were too restrictive. Nina (no diagnosis) gives an example:

*I have heard about people who go to a homeopath with one small problem, and then they tell you that you react to 30 foods (…) So I avoided this [going to homeopaths].*

Interviewees also reported going through individual processes of reorientation, which resulted in the realization that they had to put in time and effort to manage their FH. Further, interviewees described individual, emotion-focused strategies that made it easier to live with the restricted diet, like comparing their situation with that of others who were worse-off, or changing their focus from food to other aspects of their life.

Interviewees who had persons competent in FH in their close network clearly took advantage of this. These interviewees received advice about how to ensure proper nourishment despite the restricted diet. Furthermore, they could ask these network members why new symptoms had appeared and immediately receive an answer, while others spent much more time finding relevant information. Carina (celiac disease and lactose intolerance) gave an example of how family can contribute to clarifying which foods cause symptoms:

*But 2 years ago I started to have stomach trouble (…) and I never got well. And my [family member], who is lactose intolerant, said I should try to remove lactose (...) I did, and I got well.*

Scarcity of the above-mentioned individual resources, combined with a complex FH and little help from others, seemed to make the work of managing FH challenging, including finding out which foods caused symptoms and what foods and dishes to eat. Further, those who were unsure about which foods caused symptoms did not implement a restricted diet as consistently as those who were sure about this. Some interviewees also described a reduced ability to critically assess the advice of others, which led to a diet that was a mixture of FH restrictions and other dietary restrictions, which may lead to unnecessary restrictions.

Some interviewees mentioned explicitly how resource scarcity influenced the management of their FH. One example is Mary (food allergies) who said that, because of her lack of energy, it took her a long time to change her diet and find concrete dishes she could eat. Moreover, she often did not have the energy to make varied dishes:

*Sometimes I just have to go through the hassle [of making time-consuming dishes] (…) but I do not often have the energy. Usually I make dishes that do not require much effort. (…) So it is the same few dishes.*

### External factors that influenced self-management work

The interviews indicated that external factors also influenced self-management work. Some participants reported that they probably had FH for years or decades, but since it was not something they had previously thought about, the symptoms remained unexplained for years. Increased public awareness of FH in the last years contributed to the thought that their symptoms might be caused by FH, which for some was confirmed by conventional medicine. This also led to a restricted diet, and the reduction or removal of symptoms. Thus, the awareness of the phenomenon of FH could be perceived as crucial to carrying out self-management work. The participants also said that other reasons for dieting had received increased attention, and their FH was associated and mixed together with these other diets. Some mentioned that their FH was met with disbelief and criticism; that it was not taken seriously, was perceived as a fad, was linked to hysteria or stress, or was seen as a psychological problem, and participants found this to be stigmatizing.

In general, the interviewees communicated the attitude that meals should be shared, and some expressed the desire not to bother those who made food for them with their demanding restrictions. Some said they wanted to eat what they were offered, just like the others at the table, and some mentioned that they were excluded from certain social situations because of their restricted diet. In short, some interviewees did not want to break with the social expectations of the meal; instead they wanted to take part in the meal, and some interviewees ate foods they could not tolerate to avoid breaking with these expectations. However, this only applied to those with fairly weak reactions to foods. Helen (allergies) is an example of this:

“So I don’t mention [the FH], when I am at restaurants. There I eat foods that I would not eat at home.”

Participants also described increased knowledge of FH, which made self-management work easier. However, the interviewees reported varying knowledge levels among staff in restaurants and cafes, which required the participants to assess whether the person they were interacting with had sufficient competence.

The selection of foods and dishes can also be seen as both facilitating and complicating the work of managing FH. Participants with a hypersensitivity to gluten, wheat, or lactose saw the increased selection of gluten- or lactose-free goods as an advantage. Some also found it advantageous that international dishes with less milk and gluten had entered the Norwegian diet. However, the introduction of new foods in the Norwegian diet gave some interviewees new allergies, and some pointed to an increased tendency to put ‘everything in everything’, which made it more difficult to find out whether the foods they could not tolerate where included in dishes. Some mentioned that increased labeling was an advantage, although some also mentioned examples of over-labeling. Participants mentioned that courses, information, and counseling organized by patient organizations facilitated the work of managing FH. They also mentioned that carrying out a restricted diet was costly, but none said that this stopped them from implementing the diet.

We found no clear relationship between education level and the resources and self-management described in this small sample. However, competing priorities were a factor that made the self-management work difficult for some, especially the priorities of work and small children. Some interviewees expressed that dealing with work and children made it difficult to do the work of finding out what foods they could not tolerate, what concrete foods and dishes to eat, or to implement a varied diet. Frida (possible irritable bowel syndrome), who has a very restricted FODMAP diet, gave an example of how reintroducing foods can cause significant symptoms that are incompatible with work and taking care of children, and thus her diet remains very restricted:

*According to the (FODMAP) diet, you are supposed to reintroduce [foods], but you have to have an ocean of time to do this, which I do not have.*

## Discussion

The interviewees did self-management work because they were highly motivated, and this motivation was expressed through, among other things, being continuously alert to avoid foods they did not tolerate. Important motivators included the uncomfortable symptoms that resulted from consuming some foods, which had negative consequences on their energy, work, and family, or could bring shame or embarrassment.

Individual resources also played a key role in self-management work. Important individual resources were the ability to critically assess advice from others and chose what was relevant, and the realization that it would take time and effort to manage FH. Other individual resources were a broad range of problem- and emotion-focused strategies. Those who had a social network that contained people with relevant knowledge clearly took advantage of this. They received advice about how to ensure a nutritious diet despite the restrictions, and it took them less time to find out which foods caused symptoms. Scarcity of individual and social resources seemed to make it more challenging to do self-management work, and to find out which foods caused symptoms. Lack of competence about what foods that caused symptoms reduced participants’ motivation to consistently implement a restricted diet.

One external factor that was important for the management of FH was the increased public awareness of these conditions. Hindrances to this management were competing priorities, wanting to take part in meals like everyone else, and the stigma related to having a special diet. However, the economic costs of a special diet did not hinder participants from implementing it.

Many participants described uncomfortable symptoms, such as gastrointestinal symptoms. For some, these symptoms appeared a short time after they ingested adverse foods. The wish to avoid these symptoms seemed to be an important motivator for persons with FH to conduct self-management work. This is in contrast to other studies that reported that lack of immediate symptoms reduces motivation to conduct the work of managing a chronic condition [22, 23].

The participants also described that the symptoms could have negative consequences on their lives, family, and work, which they wanted to avoid. The wish to stay healthy and take care of family could be seen as intrinsic aspirations, which produce high-quality motivation according to SDT [6]. This is also in line with COR theory, which suggests that people strive to take care of and retrieve the things that they centrally value, such as health, work, and family [9]. In addition to this individual motivation to retrieve health, it has also been argued that, while in the past health was considered something that was given to us, the idea that health is an individual responsibility has become more and more prevalent in the last years [24, 25]. In other words, the fear of losing things that one really values may be a strong motivator, which may be supported by the expectation that one should take care of one’s own health.

Some interviewees also seemed to be motivated to do self-management work due to their wish to avoid embarrassment or shame. Giddens calls shame the ‘negative side of an individual’s motivational scheme’ [26:84], and other research has also indicated that embarrassment and shame have the potential to motivate [27]. The avoidance of shame, or doing actions to enhance one’s ego or pride, is also described in SDT as a relatively strong motivating force [28]. Thus, it is probable that persons with other gastrointestinal health conditions may be motivated to do self-management in order to avoid shame or embarrassment. On the other hand, some interviewees did eat adverse foods when they ate with others, which can be seen as an attempt to avoid the stigma that may be connected to a restricted diet. This is in line with studies that have shown that stigma or shame can be obstacles to self-management work, like sticking to a restricted diet [19].

In summary, the wish to avoid uncomfortable symptoms and their consequences were strong motivational forces. However, one may speculate whether these motivators are so strong that they sometimes lead to a diet that is too restricted. Larger access to competent diagnostic help from health services may reduce the extent of this phenomenon.

The interviewees presented individual strategies like problem-focused coping strategies, emotion-focused strategies, and realization of necessary work, which also have been described as enhancing self-management in studies of other chronic conditions [12, 29]. However, individual and social resources seemed to be more crucial for women with FH compared to other chronic conditions, and this may be related to the sparse health services available to adult persons with FH [15, 16].

One of the individual resources that interviewees had, to a larger or lesser extent, was the ability to critically assess the advice of others. Assessment like this may be important for persons with chronic conditions in general. However, this assessment may be especially challenging for persons with conditions like FH, because there are many FH conditions, as well as other reasons for having a special diet [15, 30], and these can be mixed together. Furthermore, health services have varying competencies, and conventional health services and alternative medicine can give confliction information [15, 17, 18].

This study showed that having knowledge about which foods caused symptoms was an important motivator for consistently sticking to a restricted diet. This is in accordance with SDT, which emphasizes that competence is important for high-quality motivation [7]. Furthermore, those who had sufficient individual and social resources seemed to be able to acquire this competence more easily, which is in accordance with COR theory [11]. This may also apply to other groups of people with chronic conditions: available individual and social resources influence whether they gain knowledge, which in turn influences their degree of motivation to do self-management work. Thus, resources may enhance self-management work both directly and indirectly, via increased access to competence that enhances motivation.

In agreement with previous studies [29], a factor that clearly influenced participants’ capacity to do self-management work was competing priorities (such as work and small children). This indicates that not only the amount of resources, but also to what degree these resources are available, influence one’s capacity to do self-management work.

Using Hobfoll’s [9] terms, material, individual, and social resources can be seen as ‘resource caravans’, while the environmental conditions that facilitate or hinder an individual are referred to as ‘caravan passageways’. One important factor that seemed to expand caravan passageways in our study was an increased public awareness of FH. Factors that seemed to narrow the caravan passageway and make the work of managing FH more demanding were the stigma related to having a restricted diet and the social expectations related to meals. Thus, while other studies reported that ‘caravan passageways’ included factors like safety, school quality, and relatively tangible conditions [9], in our study caravan passageways were described as being related to attitudes, perceptions, and ways of thinking about food and FH.

According to SDT, it is not only competence, but also autonomy and relatedness that are important for high-quality motivation [7, 8]. The interviewees presented a relatively high degree of autonomy, partly because they assessed and chose the advice that they found most useful. However, the fact that some interviewees chose to eat adverse foods may be related not only to a wish to avoid stigma, but also to a need to be part of the meal like everyone else at the table, which can be seen as ensuring the basic need of relatedness. Breaking with the expectations of the meal may obstruct the bonding and intimacy that takes place when sharing a meal [19, 31], and may thus threaten relatedness. The wish to attend to one’s basic needs probably affects self-management work in other patient groups as well. For example, receiving a self-management arrangement from health services may reduce a patient’s feeling of autonomy. Further, other conditions that require a diet, such as diabetes, may threaten the need for relatedness.

As indicated, some interviewees had the individual and social resources necessary to manage their FH, and thus hinder a negative resource cycle [11]. Others did not have these resources, and/or competing priorities kept them from doing self-management work. Based on this, one may conclude that some persons with FH do not need help from health services, while others may benefit from such help. Help from health services should be based on SDT – principles ensuring competence, relatedness, and autonomy [7]. Relatedness can be ensured through showing understanding and respect. For women with FH, one important competence is knowing what foods to avoid and what foods and dishes to eat.

As described, some participants had the resources necessary to manage their FH. However, management of FH required that participants were constantly alert, and participants also experienced negative reactions towards their restricted diet from others. Studies on persons with food allergies and their families indicate that both of these factors can compromise quality of life [32, 33]. This indicates that even though some persons are able to manage their FH successfully and maintain their health relatively well, they may still experience a reduced quality of life because they have to be constantly alert and deal with negative reactions from others.

One limitation of the study may be that the interviews were designed to focus primarily on illuminating the nature of the work that goes into managing FH. Thus, some information, for example on motivation and resources used, may not have come forth. Further, the limitation to women aged 39–67 removed the opportunity to illuminate what makes men and younger women with FH do self-management work.

One aspect which can be seen as a weakness with the study, and which have to be taken into consideration when reading the present study, is the fact that the sample had a considerably higher education level than the average Norwegian population [34]. This high education level indicates that the sample had access to more resources than the average population [11], including resources that can be used to manage FH. One cannot rule out that a sample of women with a lower education level may have revealed somewhat different findings. For example, none of the participants reported economical costs as a hindrance to implementing a restricted diet. However, this finding may not necessarily apply to other samples.

## Conclusions

The analysis showed that women with FH carried out the work of managing their condition because they were highly motivated. The wish to avoid uncomfortable symptoms, as well as the wish to avoid the negative consequences of these symptoms, were the most important motivators. In addition, some symptoms led to shameful experiences, and the wish to avoid these experiences can be motivating. Further, the analysis showed that self-management was largely dependent on the person’s individual and social resources. This may be a result of sparse health services, conflicting information, and mixing FH together with other reasons for having a special diet. Some women with FH had the individual and social resources necessary to manage their conditions, and thus may not need health services. Others may not have the necessary resources, or may have significant competing priorities, and these individuals may need help from health services. This help may ensure that the basic needs of competence and relatedness are covered. More concretely, improving competence may entail assisting women in determining which foods cause symptoms, which may contribute to avoiding unnecessary restrictions. It may also include assistance in find out what foods and dishes to eat, and assistance in ensuring a nutritious diet. Improving relatedness, through showing understanding and respect, may compensate for any loss of relatedness that people with FH may experience in relation to meals.

Covering the basic needs of competence and relatedness may also contribute to self-management among persons with other chronic conditions. On the other hand, a person’s wish to fulfil these basic needs can become an obstacle to self-management work. For example, patients may break with recommended diets to take part in the relatedness offered by sharing a meal.

The wish to avoid uncomfortable symptoms will probably motivate people with chronic conditions in general to conduct self-management work. However, this indicates that those without immediate, uncomfortable symptoms may have lower motivation. The wish to avoid shame can be a general motivator to conduct self-management work, but it can also be an obstacle to this work. Individual and social resources increase a person’s capacity to do self-management work directly and indirectly, because people with these resources are able to gain knowledge that increases their motivation.

## Data Availability

The qualitative interviews cannot be shared, since participant consent for this was not obtained.

## Abbreviations

COR theory: Conservation of resources theory
FH: Food hypersensitivity
REC: Regional Committee for Medical and Health Research Ethics
SDT: Self-Determination theory
